# Hybrids of 1,4-Naphthoquinone with Thymidine Derivatives: Synthesis, Anticancer Activity, and Molecular Docking Study

**DOI:** 10.3390/molecules28186644

**Published:** 2023-09-15

**Authors:** Monika Kadela-Tomanek, Kamil Krzykawski, Adrianna Halama, Robert Kubina

**Affiliations:** 1 Department of Organic Chemistry, Faculty of Pharmaceutical Sciences in Sosnowiec, Medical University of Silesia, Katowice, 4 Jagiellońska Str., 41-200 Sosnowiec, Poland; 2 Silesia LabMed, Centre for Research and Implementation, Medical University of Silesia in Katowice, 18 Medyków Str., 40-752 Katowice, Poland; kamil.krzykawski@sum.edu.pl (K.K.); adrianna.halama@sum.edu.pl (A.H.); rkubina@sum.edu.pl (R.K.); 3 Department of Pathology, Faculty of Pharmaceutical Sciences in Sosnowiec, Medical University of Silesia in Katowice, Ostrogórska 30, 41-200 Sosnowiec, Poland

**Keywords:** 1,4-naphthoquinone, AZT, apoptotic, head and neck cancers

## Abstract

One of the most essential health problems is cancer, the first or second cause of death worldwide. Head and neck cancers are hard to detect due to non-specific symptoms. The treatment often relies on a combination of radio and chemotherapy. For this reason, the research of new anticancer compounds is fundamental. The natural and synthetic compounds with 1,4-naphthoquinone scaffold is characterized by high anticancer activity. The study aimed to evaluate the synthesis and anticancer activity of hybrids 1,4-naphthoquinone with thymidine derivatives. The series of compounds allows us to check the influence of the substituent in the C3′ position of the thymidine moiety on the cytotoxicity against squamous cancer cell lines (SCC-9 and SCC-25) and submandibular gland cancer (A-253). An annexin V/propidium iodide (PI) co-staining assay shows that derivatives cause the apoptotic in SCC-25 and A-253 cell lines. The molecular docking study examined the interaction between the active site of the BCL-2 protein and the hybrids.

## 1. Introduction

Cancer is a major health problem worldwide, with the World Health Organization (WHO) estimating it to be the first or second leading cause of death under 70 in 112 of 183 countries [1]. The COVID-19 pandemic significantly reduced the possibilities of the detection and treatment of cancer. A statistical analysis of the impact of the pandemic on human health around the world will take several years due to data sharing issues. For these reasons, the reliable data on cancer morality are currently available until 2019 only [2]. According to the GLOBOCAN report, in 2020, there were over 19 million new cases of cancer and over 10 million deaths due to cancer. An estimated 53% of cancer deaths occur in Asia, where 59.5% of the world’s population lives. Europe accounts for almost 20% of cancer deaths, where only 9.7% of the world’s population live [1]. More than 1 million new cases have been diagnosed with head and neck cancers alone. These types of cancer are hard to detect due to non-specific symptoms [3]. Surgery is often impossible, so the therapy relies on a combination of radiotherapy and chemotherapy [4,5].

**Figure 1:**
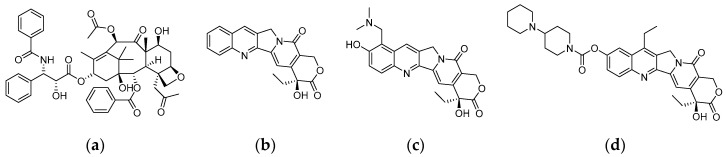
The chemical structure of (**a**) paclitaxel, (**b**) camptothecin, (**c**) topotecan, and (**d**) irinotecan.

For many years, natural substances and their semi-synthetic derivatives have been used in oncological therapy [6,7]. Paclitaxel, first extracted from the stem bark of Taxus brevifolia, is used in treating different types of cancers, such as breast, ovarian, lungs, and the head and neck (Figure 1a) [8,9]. The campthothecin, isolated from the bark of *Camptotheca acuminata*, exhibited high anticancer activity against many human tumors (Figure 1b). Unfortunately, the clinical trial was stopped due to its high toxicity. The structure–activity relationship analysis shows that modifying quinoline moiety produces a semi-synthetic compound with better water solubility and less toxicity [10]. Nowadays, two semi-synthetic derivatives, such as topotecan and irinotecan, are used to treat colon, ovarian, and lung cancers (Figure 1c,d) [6,10].

**Figure 2:**
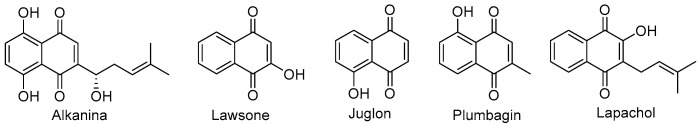
Chemical structures of natural 1,4-naphthoquinone compounds.

The naphthoquinone compounds are widely distributed as the second metabolites of plants, marine invertebrates, fungi, and bacteria [11,12]. A typical skeleton of naphthoquinone includes 1,4-naphthoquinone, 1,2-naphthoquinone, and 2,6-naphthoquinone. The most common arrangement is 1,4-naphthoquinone, which occurs in natural substances such as alkannin, lawsone, juglone, plumbagin, and lapachol (Figure 2) [13,14,15].

Compounds containing the 1,4-naphthoquinone moiety are used as dyes due to their color. For example, red-orange lawsone is used as natural hair and skin dyes, while alkanina is used in cosmetics as red pigment. Moreover, the natural 1,4-naphtoquinone substances exhibited a broad spectrum of activity, such as anticancer, antibacterial, antiviral, and antimalarial. The low solubility and toxicity of natural compounds caused us to obtain synthetic compounds with 1,4-quinolinedione scaffold [6,13,16,17]. Modifying 1,4-naphthoquinone moiety at the C2 and C3 positions leads to obtaining a new compound with high biological activity. The pharmacokinetic research showed that the synthetic derivatives exhibit good solubility and bioavailability [18,19,20,21,22].

Nucleoside and its analogues exhibit anticancer, antiviral, and antibacterial activity. The 3′-azido-3′-deoxythymidine (AZT) is used to treat acquired immunodeficiency syndrome (AIDS) [23,24,25,26]. In the last year, it was shown that AZT in combination with cisplatin, methotrexate, or 5-fluorouracil has high anticancer activity against advanced colon cancer [27,28]. Combining the 3′-azido-3′-deoxythymidine skeleton with the biological activity compound leads to new hybrids with high biological activity. The AZT moiety’s introduction affects the compounds’ bioavailability and solubility [29,30,31,32,33,34].

Our study aimed to obtain hybrids of 1,4-quinolinedione with thymidine derivatives. The anticancer activity of new compounds has been demonstrated against three human cancer cells lines. Furthermore, apoptosis was analyzed using an Annexin V/Propidium Iodide apoptosis. The annexin-V/propidium iodide method is a standard flow cytometric method for the multiparametric analysis of cells in apoptosis. The research was supplemented via a molecular docking study to clarify the reaction between the hybrids and the BCL-2 protein.

## 2. Results and Discussion

### 2.1. Chemistry

**Scheme 1:**
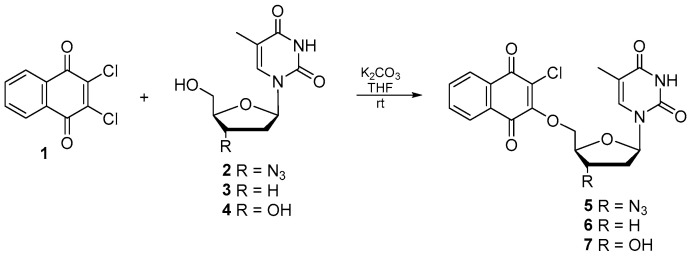
The synthesis route of thymidine derivatives **5**–**7**.

According to the literature, the 2,3-dichloro-1,4-naphthoquinone **1** is most often used as a substrate for synthesizing the 1,4-naphthoquinone derivatives [20,35,36,37]. The reaction between compound **1** and thymidine **4** or its derivatives **2**–**3** gives the monosubstituted derivatives **5**–**7** (Scheme 1).

The crude products were purified via column chromatography. The synthesis yield was high, varying within the 59-77% range. The chemical structure of compounds **5**–**7** was determined via ^1^H and ^13^C NMR, IR and HR-MS spectroscopy. The detailed analysis of the ^1^H and ^13^C NMR spectra of derivative **5** was based on the HSQC (Heteronuclear Single Quantum Coherence) and HMBC (Heteronuclear Multiple Bond Correlation) experiments (Figures S1 and S2). The numbering of atoms and the correlation between the carbon and hydrogen atoms are presented in Table S1. In the ^1^H NMR spectra, the protons of the 1,4-naphthoquinone and the 3′-azido-3-deoxythymidine moieties are observed in the range from 8.10 ppm to 7.84 ppm and from 7.56 ppm to 1.78 ppm, respectively. Based on the HSQC and HMBC correlation spectra, the signal of the carbon atoms were identified. The HMBC spectrum shows that the C3 (δ_C_ 156.6 ppm) atom of the 1,4-naphthoquinone scaffold correlates with the proton at the C5′(δ_H_ 4.76 ppm) position of the thymidine moiety. In contrast, the carbon atom at the C2 (δ_C_ 127.5 ppm) position of the 1,4-naphthoquinone scaffold does not correlate with the other protons.

This series of compounds **5**–**7** allows us to examine the influence of the substituent at the C3’ position of the thymidine moiety on the selected physicochemical properties of the tested derivatives.

The physicochemical properties, such as lipophilicity (log P), solubility (S), the number of donors (nHD) and acceptors (nHA) of hydrogen bonds, the number of rotatable bonds (nRT), molecular weight (MW), and the topological polar surface area (TPSA) were determined using the SwissADMET platform [38]. According to Lipinski and Veber rules, these parameters determine the bioavailability of compounds. The results are presented in Table 1.

As seen in Table 1, the substituent at the C3′ position of the thymidine scaffold determines the physiochemical properties. All tested compounds show solubility in water. Replacement of the hydroxyl group with the hydrogen atom (**6**) or the azide group (**5**) reduces the water solubility. Comparing the S value shows that compound **7** has more than 15 times and 4 times better water solubility than derivatives **5** and **6**, respectively. Lipophilicity determines the solubility of a substance in a non-polar solvent. A higher log P value means that the compound is more soluble in a non-polar solvent than in a polar one [39]. The tested hybrids were characterized by low log P values, which means that they were moderately soluble in non-polar solvents. The introduction of the hydroxyl group at the C3′ position reduces the lipophilicity. The replacement of the hydrogen atom (**6**) by the azide group (**5**) slightly increases the lipophilicity. The topological polar surface area (TPSA) depends on the number and type of heteroatoms, such as the oxygen and nitrogen atoms, present in the molecular structure of the compound [40]. The introduction of a group containing a heteroatom (compounds **5** and **7**) increases the TPSA, and its sizes are 1.5 times and 1.2 times higher than for derivative **6**. Hybrids **5**–**7** meets all of Lipinski’s rule criteria, such as MW being less than 500, log P being less than 5, and A and HD being less than 10 and 5, respectively [39]. According to the Veber criterion, compounds **6** and **7** meets the rule regarding that TPSA must be less than 400 Å and nRT must be less than 10 [40]. The results show that the tested compounds should be characterized by good oral bioavailability.

### 2.2. Biological Study

The cytotoxicity of newly synthesized compounds **5**–**7** and 2,3-dichloro-1,4-naphthoquinone **1** was tested against squamous cancer cell lines (SCC-9 and SCC-25), submandibular gland cancer (A-253), and a normal epithelial (Beas-2B) cell line. An MTT dye reduction assay assessed cell viability.

**Figure 3:**
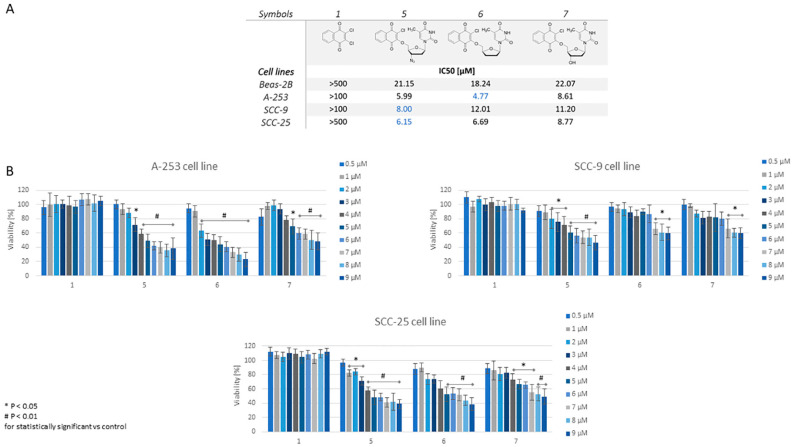
Cytotoxicity effect of compounds **1** and **5**–**7** against A-253, SCC-9, and SCC-25 cancer cell lines and normal epithelial (Beas-2B) cell line. (**A**) The calculated IC_50_ values of tested derivatives for 48 h cell incubations are presented in the table. The IC_50_ value was calculated with ED50plus, which is a tool for pharmacological analysis which allows us to create and analyze dose-response curves. (**B**) Visual graphs presenting the dose- and time-dependent decrease in cell viability of the test lines after treatment with tested derivatives for 48 h. The blue color means the lowest IC_50_ value against tested line.

Cell viability was assessed via an MTT dye reduction assay and the IC_50_ for compounds **1** and **5**–**7** was determined from the dose-response curves (Figure 3).

Compound **1**, the negative control, shows low cytotoxicity against the tested cell lines, and the IC_50_ is higher than 100 µM for all lines. In the series of tested hybrids **5**–**7,** the lowest activity against all tested cell lines shows thymidine derivative **7**. In the tested hybrids **5**–**7** group, compound **5** shows the highest cytotoxicity against squamous cancer cell lines. The IC_50_ equals 6.15 µM and 8.00 µM for SCC-25 and SCC-9, respectively. Replacement of the azide group at the C3′ position of the thymidine moiety by the hydrogen atom or hydroxyl group reduces the activity against these cell lines. The 2-chloro-3-(3′-deoxythymidine)-1,4-naphtoquinone **6** shows the lowest value of IC_50_ against the salivary gland adenocarcinoma (A-253) line, which is 4.77 µM. Comparing the cytotoxicity against the A-253 cell line shows that the activity order is **6** > **5** > **7**.

The toxicity of compounds **1** and **5**–**7** was determined by evaluating the activity against the normal epithelial (Beas-2B) cell line. Compound **1** did not show any activity against the normal cell line. Hybrids **5**–**7** exhibited lower activity against the Beas-2B cell line than the cancer cell lines. Moreover, the selectivity index (SI) was calculated (Table S2). Derivatives **5**–**7** show the highest selectivity index (SI = 2.56–3.82) against the A-253 cell line. According to the classification of Weerapreeyakul, the tested derivatives **5**–**7** are categorized as potential anticancer compounds [41].

An annexin V/propidium iodide (PI) co-staining assay was performed to determine the mode of cell death caused by compounds **5**–**7**. This test differentiates between necrotic (PI+) and apoptotic cell death, divided into early apoptotic [PI(-) Annexin-V (+)] and late apoptotic [PI(+) Annexin-V (+)].

**Figure 4:**
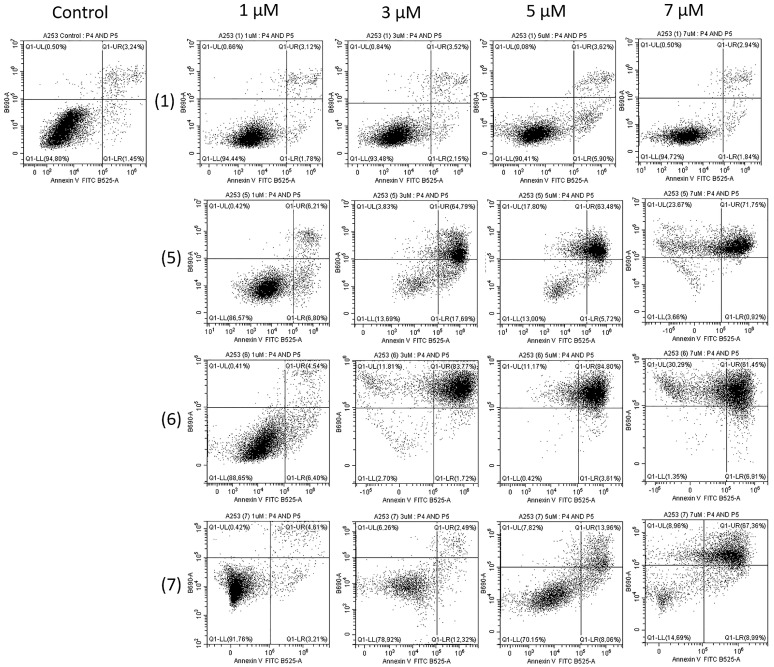
Representative scatter plots differentiating apoptotic and necrotic from viable cells based on annexin V-FITC and PI staining after 48 h incubation with compound **1** and derivatives **5**–**7** and flow cytometric analysis. (Q1) necrotic cells; (Q2) late apoptotic cells; (Q3) early apoptotic cells; and (Q4) viable cells. The percentage of apoptotic cells in the A-253 cells groups were significantly increased compared to controls.

**Figure 5:**
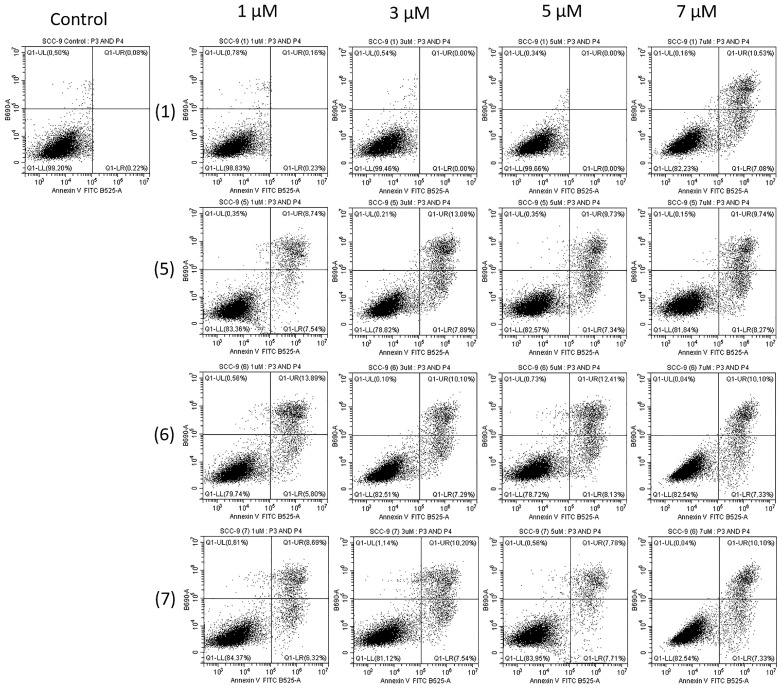
Representative scatter plots differentiating apoptotic and necrotic from viable cells based on annexin V-FITC and PI staining after 48 h incubation with compound **1** and derivatives **5**–**7** and flow cytometric analysis. (Q1) necrotic cells; (Q2) late apoptotic cells; (Q3) early apoptotic cells; and (Q4) viable cells. The percentage of apoptotic cells in the SCC-9 cells groups were significantly increased compared to controls.

**Figure 6:**
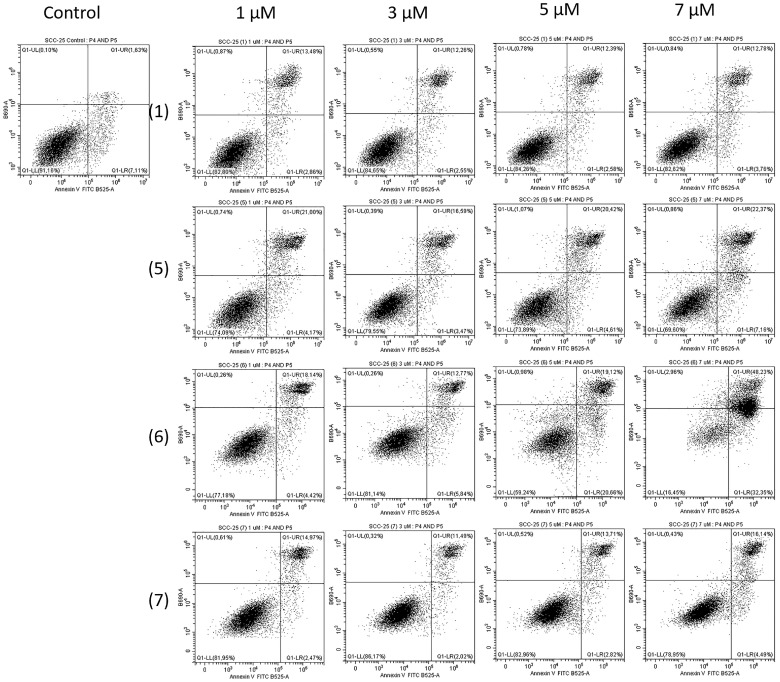
Representative scatter plots differentiating apoptotic and necrotic from viable cells based on annexin V-FITC and PI staining after 48 h incubation with compound **1** and derivatives **5**–**7** and flow cytometric analysis. (Q1) necrotic cells; (Q2) late apoptotic cells; (Q3) early apoptotic cells; and (Q4) viable cells. The percentage of apoptotic cells in the SCC-25 cells groups was significantly increased compared to controls.

Cell lines SCC-9, SCC-25, and A-253 were exposed to hybrids **5**–**7** at 1 μM, 3 μM, 5 μM, and 7 μM for 48 h, and then double stained with FITC-conjugated annexin V and propidium iodide (PI) and analyzed via flow cytometry. The results are presented in Figure 4, Figure 5 and Figure 6.

After the 48-hour reaction with the tested hybrids, it has been observed that the most sensitive line to compounds **5**–**7** is the squamous cell carcinoma of the salivary glands (A-253). The most substantial effects for this line are observed for compounds **5** and **6** at the concentration of 3 µM (Figure 4). Compound **7** causes the appearance of late apoptotic cells at the concentration of 7 µM. The obtained results are consistent with the cytotoxicity determined via the MTT test.

It is also observed that the SCC-9 line is the most resistant to the tested compounds. As seen in Figure 5, the results are similar for all tested derivatives at concentrations of 1-7 µM. From the detailed analysis, compounds **5** and **6** cause the appearance of the highest number of early apoptotic and late apoptotic cells.

In the case of the SCC-25 line, the highest pro-apoptotic activity is shown by compound **6**, causing an almost complete reduction in live cells (16.45%) and the appearance of apoptotic cells above 80% (Figure 6). In addition, compound **5** at a concentration of 7 µM reduces the number of viable cells to about 70% and increases the number of apoptotic cells to about 30%.

### 2.3. Molecular Docking

Biological studies have shown that compounds **5**–**7** cause apoptosis in squamous cancer cell lines (SCC-9 and SCC-25). The apoptosis may be caused by the inhibition of anti-apoptotic (BCL-2) proteins [42]. For this reason, we checked the interaction between the molecules and the BCL-2 protein using the AutoDock Vina program [43].

The scoring function (ΔG) value shows that hybrids **5**–**7** have a higher affinity for the BCL-2 protein than substrate **1**. The type of substituent at the C3′ position of the thymidine moiety slightly influences the binding of the ligand to the active center of the protein (Table 2).

**Figure 7:**
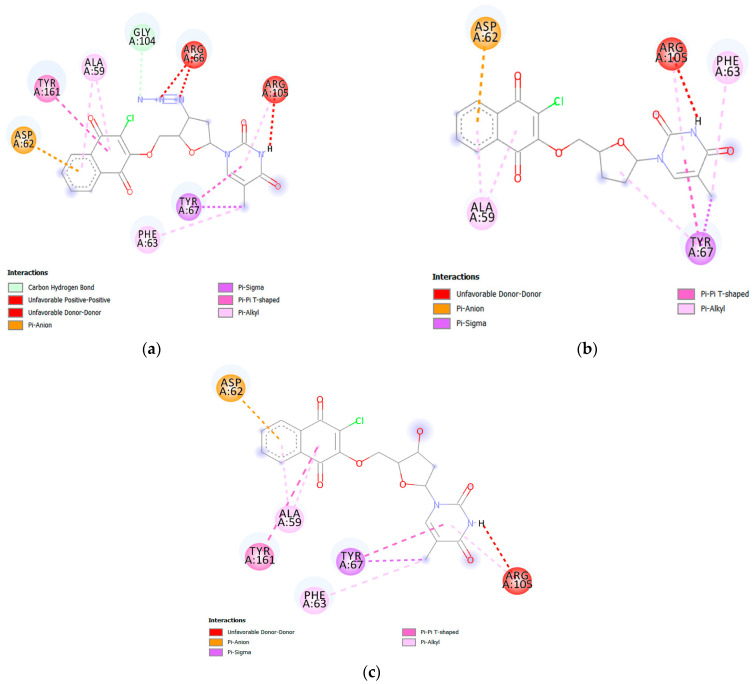
Docking pose of the BCL-2 protein complex with hybrids: (**a**) **5**; (**b**) **6**; and (**c**) **7**.

Figure 7 shows a detailed interaction analysis between ligands **5**–**7** and the BCL-2 protein. The type of bonds and their distances are presented in Table S3.

Hydrophobic and electrostatic interaction were observed in the ligand–protein complex. The 1,4-naphtoquinone creates a hydrophobic interaction with alanine (ALA59) and an electrostatic interaction with aspartic acid (ASP62). Ligands **5** and **7** also create a hydrophobic interaction with tyrosine (TYR161). The thymidine moiety interacts with tyrosine (TYR67), phenylalanine (PHE63), and arginine (ARG105). Only in the complex between ligand **5** and the protein is when an electrostatic interaction between the substituent at the C3′ position and arginine (ARG66) is observed. Additionally, the azide group creates a hydrogen bond with glycine (GLY104).

Comparing the biological activity with the molecular docking study shows that the highest activity against the SCC-25 cell line is seen for compound **5,** which creates a hydrogen bond with an active center of the protein. These results suggest that the presence of hydrogen bonds in the interaction between the ligand and the active center of the BCL-2 protein favors the occurrence of cytotoxicity.

## 3. Materials and Methods

### 3.1. Chemistry

The nuclear magnetic resonance (NMR) spectra were measured using the Bruker Avance 600 spectrometer (Bruker, Billerica, MA, USA) in methanol-d4 solvents. Chemical shifts (δ) are reported in ppm and J values in Hz. Multiplicity is designated as the doublet (d), doublet of doublets (dd), triplet (t), and multiplet (m). High-resolution mass spectral analysis (HR-MS) was recorded using the Bruker Impact II instrument (Bruker, Billerica, MA, USA). Calculation of the theoretical molecular mass of compounds was performed using “The Exact Mass Calculator, Single Isotope Version” (http://www.sisweb.com/referenc/tools/exactmass.htm (accessed on 26 June 2023); (Ringoes, NJ, USA)). Infrared spectra (IR) were performed using the IRXross spectrophotometer equipped with the attenuated total reflection (ATR) mode. Melting points were measured via the Electrothermal IA 9300 melting point apparatus.

All commercial substances were purchased in Merck (Darmstadt, Germany).

#### General Synthesis of Compounds

Thymidine **4** or its derivatives **2**–**3** (1,2 mmol) and potassium carbonate (0.138 g; 1 mmol) were dissolved in tetrahydrofuran (4 mL). The 2,3-dichloro-1,4-naphtoquinone **1** (0.227 g; 1 mmol) in tetrahydrofuran (2 mL) was added. The reaction mixture was stirred at room temperature and the TLC method monitored the reaction progress. After the entire conversion of substrate **1**, the reaction mixture was concentrated with a vacuum evaporator. The crude product was purified via column chromatography (SiO_2_, chloroform/ethanol, 10:1, v/v) to give the pure compounds **5**–**7**.

*2-Chloro-3-(3′-azido-3′-deoxythymidine)-1,4-naphtoquinone* **5***:* Yield 77%; mp 88-90 °C; ^1^H NMR (600 MHz, CD_3_OD) δ, ppm: 1.82 (d, J = 1.2 Hz, 3H, H7t), 2.45 (m, 1H, H2’t), 2.61 (m, 1H, H2’t), 4.16 (m, 1H, H3’t), 4.63 (m, 1H, H4’t), 4.76 (dd, J = 3.6 Hz, J = 12.0 Hz, 1H, H5’t), 5.06 (dd, J = 3.6 Hz, J = 12.0 Hz, 1H, H5’t), 6.18 (t, J = 6.6 Hz, 1H, H1’t), 7.56 (d, J = 1.2 Hz, 1H, H6t), 7.84 (m, 2H, H6q, H7q), 8.07 (m, 1H, H5q), 8.10 (m, 1H, H8q) (Figure S3); ^13^C NMR (150 MHz, CD_3_OD) δ, ppm: 11.1 (C7t), 36.1 (C2’t), 60.2 (C4’t), 71.8 (C5’t), 82.8 (C3’t), 84.7 (C1’t), 110.5 (C5t), 126.3 (C8q), 126.4 (C5q), 127.5 (C2q), 130.9 (C9q), 131.0 (C10q), 133.8 (C7q), 134.8 (C6q), 136.3 (C6t), 150.7 (C2t), 156.6 (C3q), 164.7 (C4t), 178.3 (C1q), 179.4 (C4q) (Figure S4); IR (ν_max_ cm^−1^, ATR): 3177–2926, 2100, 1667, 1595, 1248, 1011; ESI-HRMS *m*/*z* [M + Na]^+^ calcd for C_20_H_16_ClN_5_O_6_Na^+^ 480.0687, found 480.0688.
*2-Chloro-3-(3′-deoxythymidine)-1,4-naphtoquinone* **6**: Yield 59%; mp 115–117 °C; ^1^H NMR (600 MHz, CD_3_OD) δ, ppm: 1.84 (d, J = 1.2 Hz, 3H, H7t), 2.18 (m, 3H, H2’t, 2xH3’t), 2.23 (m, 1H, H2’t), 4.39 (m, 1H, H4’t), 4.70 (dd, J = 3.6 Hz, J = 12.0 Hz, 1H, H5’t), 5.14 (dd, J = 3.6 Hz, J = 12.0 Hz, 1H, H5’t), 6.05 (t, J = 6.6 Hz, 1H, H1’t), 7.55 (d, J = 1.2 Hz, 1H, H6t), 7.82 (m, 2H, H6q, H7q), 8.06 (m, 1H, H5q), 8.09 (m, 1H, H8q) (Figure S5); ^13^C NMR (150 MHz, CD_3_OD) δ, ppm: 11.2 (C7t), 25.0 (C3’t), 30.6 (C2’t), 56.1 (C4’t), 73.2 (C5’t), 85.6 (C1’t), 110.0 (C5t), 126.2 (C8q), 126.3 (C5q), 127.1 (C2q), 130.9 (C9q), 131.1 (C10q), 133.8 (C7q), 134.0 (C6q), 136.2 (C6t), 150.7 (C2t), 157.1 (C3q), 164.9 (C4t), 178.5 (C1q), 179.5 (C4q) (Figure S6); IR (ν_max_ cm^−1^, ATR): 3169–2828, 1697, 1661, 1593, 1246, 1009; ESI-HRMS *m*/*z* [M + Na]^+^ calcd for C_20_H_17_ClN_2_O_6_Na^+^ 439.0673, found 439.0667.
*2-Chloro-3-thymidine-1,4-naphtoquinone **7***: Yield 63%; mp 128–130 °C; ^1^H NMR (600 MHz, acetone-d6) δ, ppm: 1.78 (d, J = 1.2 Hz, 3H, H7t), 2.77 (m, 1H, 2xH2’t), 4.31 (m, 1H, H3’t), 4.73 (d, J = 2.4 Hz, 1H, H5’t), 5.06 (dd, J = 3.6 Hz, J = 12.0 Hz, 1H, H5’t), 5.96 (m, 1H, H4’t), 6.60 (m, 1H, H1’t), 7.56 (d, J = 1.2 Hz, 1H, H6t), 7.84 (m, 2H, H6q, H7q), 8.07 (m, 1H, H5q), 8.10 (m, 1H, H8q), 10.04 (s, 1H, NH) (Figure S7); ^13^C NMR (150 MHz, acetone-d6) δ, ppm: 11.4 (C7t), 38.3 (C2’t), 68.0 (C4’t), 73.6 (C5’t), 84.2 (C3’t), 84.8 (C1’t), 110.3 (C5t), 126.5 (C8q), 126.6 (C5q), 127.8 (C2q), 130.3 (C9q), 131.1 (C10q), 134.2 (C7q), 134.5 (C6q), 135.5 (C6t), 150.4 (C2t), 156.6 (C3q), 163.2 (C4t), 178.2 (C1q), 179.6 (C4q) (Figure S8); IR (ν_max_ cm^−1^, ATR): 3071–2928, 1664, 1593, 1246, 1008; ESI-HRMS *m*/*z* [M + Na]^+^ calcd for C_20_H_17_ClN_2_O_7_Na^+^ 455.0622, found 455.0608.

### 3.2. Biological Study

#### 3.2.1. Materials

Sterile dimethyl sulfoxide (DMSO cat. no. D2650) and hydrocortisone (cat. no. H6909) purchased from Merck were used. Cell culture media, fetal serum, antibiotics, PBS, and trypsin were purchased in Corning (Corning Life Science, Painted Post, NY, USA). The cell lines were from the ATCC collection.

#### 3.2.2. Compounds Treatment

The stock solutions of derivatives **5**–**7** and 2,3-dichloro-5,8-quinolinedione **1** (5 mg/mL) were prepared by dissolving compounds in DMSO and then stored and frozen at –80 °C until use, but for no longer than 14 days. Concentrations in the 0.5–15 µM range were used for the cytotoxic assay and determination of IC_50_ values. For the cytometric studies, the following concentrations of 1 µM, 3 µM, 5 µM, and 7 µM were used. An additional control consisted of cells grown in a medium containing an equivalent amount of DMSO without adding compounds.

#### 3.2.3. Cell Culture

The tongue cell cancer lines SCC-9 and SCC-25 were cultured according to the manufacturer’s instructions in the DMEM/F-12 medium (1:1 ratio) supplemented with 10% fetal bovine serum FBS; antibiotics such as penicillin (100 IU mL), streptomycin (100 µL/mL), and amphotericin B (0.25 µL/mL); and hydrocortisone (400 ng/mL). Carcinoma of the salivary glands line A-253 was cultured in McCoy’s 5a Medium modified with the addition of serum and antibiotics in the concentration given above. Normal bronchial epithelium (Beas-2B) cells were cultured in Ham’s Nutrient mixture F-12 with 10% fetal bovine serum. The cultured cells were kept at 37 °C in a steam-saturated atmosphere with 5% carbon dioxide (CO_2_) added in 75 cm^2^ bottles (PAA).

#### 3.2.4. MTT Test

The cytotoxicity of the compounds was tested via the MTT test which measures the ability of cells to reduce the 3-(4,5-dimethylthiazol-2-yl)-2,5-diphenyltetrazolium bromide. The cells were seeded in 96-well plates at 5 × 10^3^ cells/well in 100 mL of culture medium and allowed to grow for 24 h. The medium was then replaced with a medium supplemented with various concentrations of the tested compounds. The cells were incubated for 48 h at 37 °C and 5% CO_2_. Next, the medium was removed and the MTT test at a 1 mg/mL concentration was added to each well and incubated for 4 h. Next, the supernatant was removed and the water-insoluble formazan crystals were dissolved in 150 µL of DMSO. The absorbance of the samples was measured using the Varioscan Lux microplate reader (Thermo Fisher Scientific, Waltham, MA, USA). Readings were made at a wavelength of 570 nm.

#### 3.2.5. FITC Annexin V Apoptosis Detection Kit II

The cells were seeded into a 6-well culture plate at a density of 5 × 10^5^ cells/dish and incubated for 48 h at 37 °C and 5% CO_2_. After incubation, the cells were stripped and washed twice with a cold PBS buffer. The apoptotic effects were analyzed using the Annexin V Apoptosis Detection Kit II (BD Pharmingen, San Diego, CA, USA), which contains the buffer-binding Annexin V-FITC and the PI staining buffer.

Briefly, the cells were resuspended in 1 ml of binding buffer to give a concentration of 1 × 10^6^ cells/ml. Next, 100 µL of suspensions (1 × 10^5^ cells/mL) was transferred to 5 mL test tubes and the Annexin V-FITC (5 µL) and PI (5 µL) dyes were added. The mixture was incubated for 15 min at room temperature in the dark. After this time, the 400 µL binding buffer was added. Cell apoptosis was quantified using a CytoFlex Srt flow cytometer (Beckman Coulter, Miami, FL, USA) and the dedicated CytExpert Srt software Version 1.1.

#### 3.2.6. Statistical Analysis

Results are expressed as means ± SD obtained from three separate experiments performed in quadruplicate (n = 12) for the cytotoxicity. The experimental means were compared with the means of untreated cells harvested in parallel. Differences between samples incubated for 48 hours were tested for significance using one-way ANOVA Friedman’s. A p-value less than 0.05 was considered statistically significant.

### 3.3. Molecular Docking Study

A molecular docking study was carried out using the crystal structure of the human BCL-2 protein, which was collected from the Protein Data Bank (PDB) database with the PDB identifier 4IEH [44].

A molecular docking study was performed with the AutoDock Vina 1.5.6. software package [43]. The grid center of Vina docking was selected as the center of the reference ligands that accompanied the downloaded protein complex. The grid size was set to 14 Å × 14 Å × 14 Å, which is large enough to cover the entire target active site. The default values of all other parameters were used and the complexes were submitted to eight genetic algorithm runs. The results were visualized using the BIOVIA Discovery Studio 2017 software package [45].

## 4. Conclusions

Combining the 1,4-naphthoquinone moiety with the thymidine derivatives allowed us to obtain a new class of thymidine hybrids as potent anticancer agents. The 2-chloro-3-(3′-azido-3′-deoxythymidine)-1,4-naphtoquinone **5** shows the highest cytotoxicity against the squamous (SCC-25) cell cancer line. The replacement of the azide group at the C3′ position of the thymidine moiety via the hydrogen atom or the hydroxyl group reduces activity against these cell lines. An annexin V/propidium iodide (PI) co-staining assay was performed to determine the mode of cell death caused by the tested derivatives. Compound **5** at a concentration of 7 µM reduced the number of viable cells to about 70% and increased the number of apoptotic cells to about 30%. The molecular docking study was used to examine the interaction between the active site of the BCL-2 protein and the tested compounds. The results show that only ligand **5** creates hydrogen bonds with the active center of the BCL-2 protein.

## Figures and Tables

**Table 1:** The selected physicochemical parameters of compounds **5**–**7**.

Physicochemical Properties	Compound 5	Compound 6	Compound 7
MW [g/mol]	457.82	416.81	432.81
TPSA [Å]	157.21	107.46	127.69
log P	2.64	2.63	1.83
S [µM]	31.4	116	488
nHA	9	6	7
nHD	1	1	2
nRT	5	4	4

**Table 2:** Vina affinity scoring values (ΔG) [kcal/mol] for ligand **1** and **5**–**7**.

Compound	ΔG [kcal/mol]
**1**	−6.70
**5**	−8.60
**6**	−8.70
**7**	−8.00

## Data Availability

Not applicable.

## Supplementary Materials

The following supporting information can be downloaded at: https://www.mdpi.com/article/10.3390/molecules28186644/s1, Table S1. The proton–carbon correlations (HSQC and HMBC experiments) for compound 5 (δ [ppm]-chemical shift of the corresponding signals in the ^1^H NMR and ^13^C NMR spectra); Table S2: The selectivity index (SI) value for compounds 5–7; Table S3: Interaction of hybrids 5–7 with active site of BCL-2 protein; Figure S1: The ^1^H-^13^C HSQC spectrum (600 MHz, CD_3_OD) of 2-chloro-3-(3′-azide-3′-deoxythimidine)-1,4-naphthoquinone 5; Figure S2: The ^1^H-^13^C HMBC spectrum (600 MHz, CD_3_OD) of 2-chloro-3-(3′-azide-3′-deoxythimidine)-1,4-naphthoquinone 5; Figure S3: The ^1^H NMR spectrum (600 MHz, CD_3_OD) of 2-chloro-3-(3′-azide-3′-deoxythimidine)-1,4-naphthoquinone 5; Figure S4: The ^13^C NMR spectrum (150 MHz, CD_3_OD) of 2-chloro-3-(3′-azide-3′-deoxythimidine)-1,4-naphthoquinone 5; Figure S5: The ^1^H NMR spectrum (600 MHz, CD_3_OD) of 2-chloro-3-(3′-deoxythimidine)-1,4-naphthoquinone 6; Figure S6: The ^13^C NMR spectrum (150 MHz, CD_3_OD) of 2-chloro-3-(3′-deoxythimidine)-1,4-naphthoquinone 6; Figure S7: The ^1^H NMR spectrum (600 MHz, aceton-d6) of 2-chloro-3-thimidine-1,4-naphthoquinone 7; Figure S8: The ^13^C NMR spectrum (150 MHz, aceton-d6) of 2-chloro-3-thimidine-1,4-naphthoquinone 7.

## Sample Availability

Samples of compounds **1** and **5**–**7** are available from the authors.
