# Supplementary material for: Hybrids of 1,4-Naphthoquinone with Thymidine Derivatives: Synthesis, Anticancer Activity, and Molecular Docking Study
Source: Molecules. 2023 Sep 15;28(18):6644. doi: 10.3390/molecules28186644 (PMC10535307; doi:10.3390/molecules28186644)
Supplement: Supplementary file 1 [file molecules-28-06644-s001.zip › molecules-2505886-supplementary.pdf]

Supplementary Materials

Hybrids of 1,4-naphthoquinone with thymidine derivatives: synthesis, anticancer activity and molecular docking study

Monika Kadela-Tomanek, Kamil Krzykawski, Adrianna Halama and Robert Kubina

Table of Contents

Table S1. The proton-carbon correlations (HSQC and HMBC experiments) for compound **5** (δ [ppm]-chemical shift of the corresponding signals in the ^{1}H NMR and ^{13}C NMR spectra). 2

Table S2: The selectivity index (SI) value for compounds **5-7**. 3

Table S3: Interaction of hybrids **5-7** with active site of BCL-2 protein. 3

Figure S1: The ^{1}H-^{13}C HSQC spectrum (600 MHz, CD_3OD) of 2-chloro-3-(3′-azide-3′-deoxythymidine)-1,4-naphthoquinone **5**. 4

Figure S2: The ^{1}H-^{13}C HMBC spectrum (600 MHz, CD_3OD) of 2-chloro-3-(3′-azide-3′-deoxythymidine)-1,4-naphthoquinone **5**. 4

Figure S3: The ^{1}H NMR spectrum (600 MHz, CD_3OD) of 2-chloro-3-(3′-azide-3′-deoxythymidine)-1,4-naphthoquinone **5**. 5

Figure S4: The ^{13}C NMR spectrum (150 MHz, CD_3OD) of 2-chloro-3-(3′-azide-3′-deoxythymidine)-1,4-naphthoquinone **5**. 5

Figure S5: The ^{1}H NMR spectrum (600 MHz, CD_3OD) of 2-chloro-3-(3′-deoxythymidine)-1,4-naphthoquinone **6**. 6

Figure S6: The ^{13}C NMR spectrum (150 MHz, CD_3OD) of 2-chloro-3-(3′-deoxythymidine)-1,4-naphthoquinone **6**. 6

Figure S7: The ^{1}H NMR spectrum (600 MHz, aceton-d6) of 2-chloro-3-thymidine-1,4-naphthoquinone **7**. 7

Figure S8: The ^{13}C NMR spectrum (150 MHz, aceton-d6) of 2-chloro-3-thymidine-1,4-naphthoquinone **7**. 7

Table S1. The proton-carbon correlations (HSQC and HMBC experiments) for compound **5** (δ [ppm]-chemical shift of the corresponding signals in the ^{1}H NMR and ^{13}C NMR spectra).

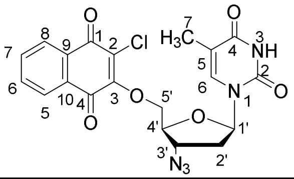

Proton	^{1}H NMR δ [ppm]	Carbon	^{13}C NMR δ [ppm]	HSQC	HMBC
-	-	C1q	178.3	-	H8q (8.10)-C1q (178.3)
-	-	C2q	127.5	-	-
-	-	C3q	156.6	-	H5′t (4.76)-C3q (156.6)
-	-	C4q	179.4	-	H5q (8.07)-C4q (179.4)
H5q	8.07	C5q	126.4	H5q (8.07)-C5q (126.4)	H5q (8.07)-C6q (134.1)
					H5q (8.07)-C10q (130.9)
H6q	7.84	C6q	134.1	H6q (7.84)-C6q (134.1)	H6q (7.84)-C5q (126.4)
H7q	7.84	C7q	133.8	H7q (7.84)-C7q (133.8)	H7q (7.84)-C8q (126.3)
H8q	8.10	C8q	126.3	H8q (8.10)-C8q (126.3)	H8q (8.10)- C7q (133.8)
					H8q (8.10)-C9q (131.0)
		C10q	131.0	-	H5q (8.07)-C10q (130.9)
		C9q	130.9	-	H8q (8.10)-C9q (131.0)
-	-	C2t	150.7	-	H6t (7.56)-C2t (150.7)
					H1′t (6.18)-C2t (150.7)
-	-	C4t	164.7	-	H6t (7.56)-C4t (164.7)
					H7t (1.82)-C4t (164.7)
-	-	C5t	110.5	-	H6t (7.56)-C5t (110.5)
					H7t (1.82)-C5t (110.5)
H6t	7.56	C6t	136.3	H6t (7.56)-C6t (136.3)	H6t (7.56)-C4t (164.7)
					H6t (7.56)-C2t (150.7)
					H6t (7.56)-C5t (110.5)
					H6t (7.56)-C1′t (84.7)
					H6t (7.56)-C7t (11.1)
H7t	1.82	C7t	11.1	H7t (1.82)-C7t (11.1)	H7t (1.82)-C4t (164.7)
					H7t (1.82)-C6t (136.3)
					H7t (1.82)-C5t (110.5)
H1′t	6.18	C1′t	84.7	H1′t (6.18)-C1′t (84.7)	H1′t (6.18)-C2t (150.7)
					H1′t (6.18)-C6t (136.3)
					H1′t (6.18)-C4′t (60.2)
H2′t	2.61	C2′t	36.1	H2′t (2.61)-C2′t (36.1)	H2′t (2.61)-C3′t (82.8)
	2.45			H2′t (2.45)-C2′t (36.1)	H2′t (2.61)-C4′t (60.2)
					H2′t (2.45)-C3′t (82.8)
					H2′t (2.45)-C4′t (60.2)
H3′t	4.16	C3′t	82.8	H3′t (4.16)-C3′t (82.8)	H3′t (4.16)-C5′t (71.8)
					H3′t (4.16)-C4′t (60.2)
H4′t	4.63	C4′t	60.2	H4′t (4.63)-C4′t (60.2)	H4′t (4.63)-C1′t (84.7)
					H4′t (4.63)-C3′t (82.8)
					H4′t (4.63)-C4′t (60.2)
H5′t	5.06	C5′t	71.8	H5′t (5.06)-C5′t (71.8)	H5′t (5.06)-C3′t (82.8)
	4.76			H5′t (4.76)-C5′t (71.8)	H5′t (5.06)-C4′t (60.2)
					H5′t (4.76)-C3q (156.6)
					H5′t (4.76)-C4′t (60.2)

Table S2. The selectivity index (SI) value for compounds **5-7**.

SI	**5**	**6**	**7**
A-253	3,53	3,82	2,56
SCC-9	2,64	1,52	1,97
SCC-25	3,44	2,73	2,52

Table S3. Interaction of hybrids **5-7** with active site of BCL-2 protein.

Ligand	H-bonding residues and length (Å)	Electrostatic interaction residues and length (Å)	π-interaction residues and length (Å)
**5**	GLY104 (3.740)	ASP62 (3.856)<br>ARG66 (4.302, 4.984)<br>ARG105 (2.160)	TYR67 (3.624, 5.350)<br>TYR161 (5.718)<br>PHE63 (4.966)<br>ALA59 (4.550, 5.277)<br>ARG105 (5.090)
**7**	-	ASP62 (3.980)<br>ARG105 (2.236)	TYR67 (3.704, 5.335, 5.256)<br>PHE63 (5.062)<br>ALA59 (4.479, 4.608)<br>ARG105 (5.107)
**8**	-	ASP62 (3.966)<br>ARG105 (2.464)	TYR67 (3.708, 5.282)<br>TYR161 (5.818)<br>PHE63 (5.071)<br>ALA59 (4.469, 4.720)<br>ARG105 (5.146)

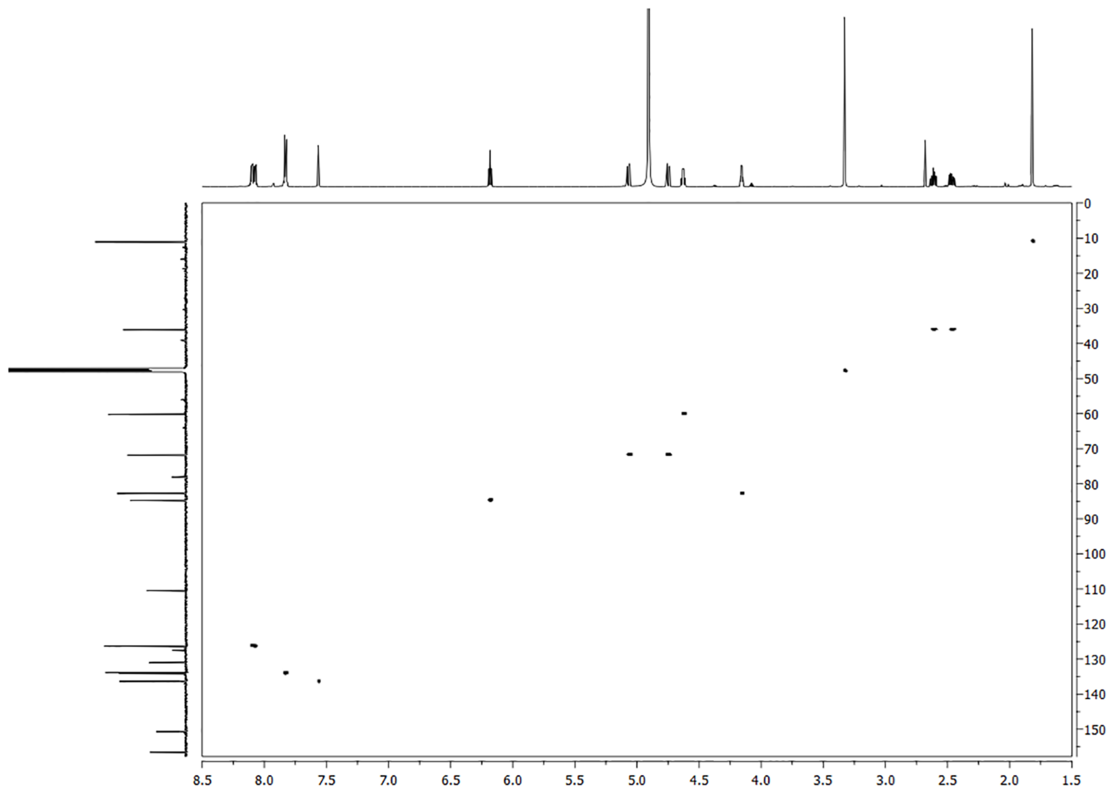

Figure S1. The ^{1}H-^{13}C HSQC spectrum (600 MHz, CD_3OD) of 2-chloro-3-(3′-azide-3′-deoxythymidine)-1,4-naphthoquinone **5**.

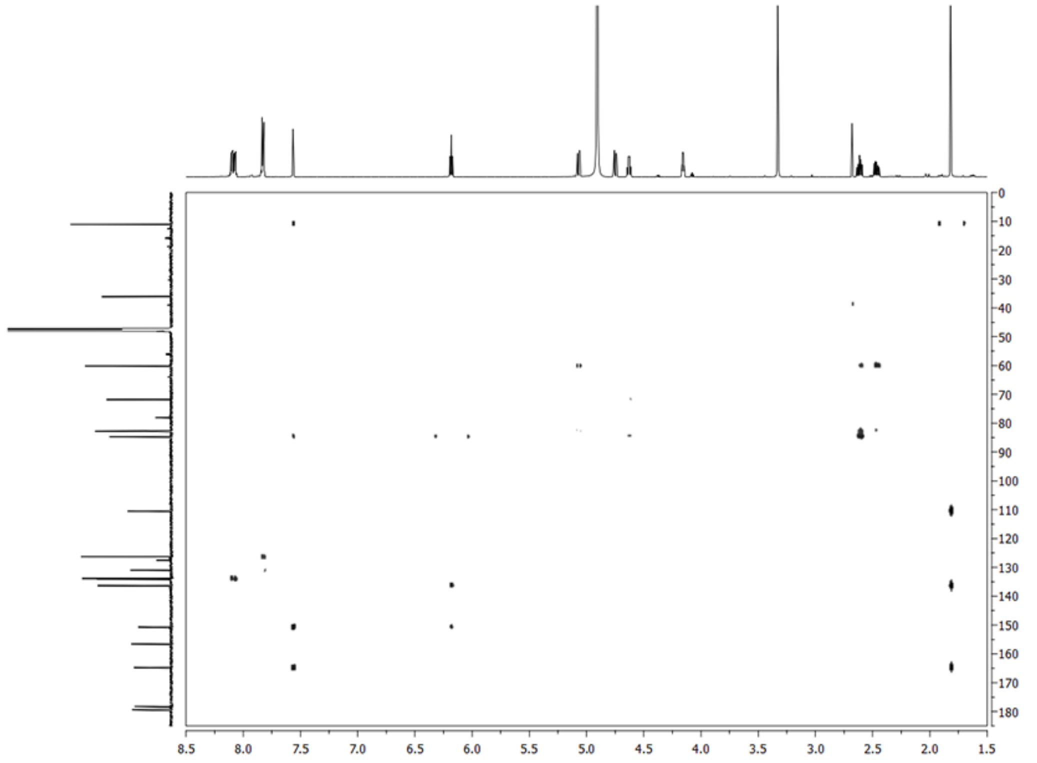

Figure S2. The ^{1}H-^{13}C HMBC spectrum (600 MHz, CD_3OD) of 2-chloro-3-(3′-azide-3′-deoxythymidine)-1,4-naphthoquinone **5**.

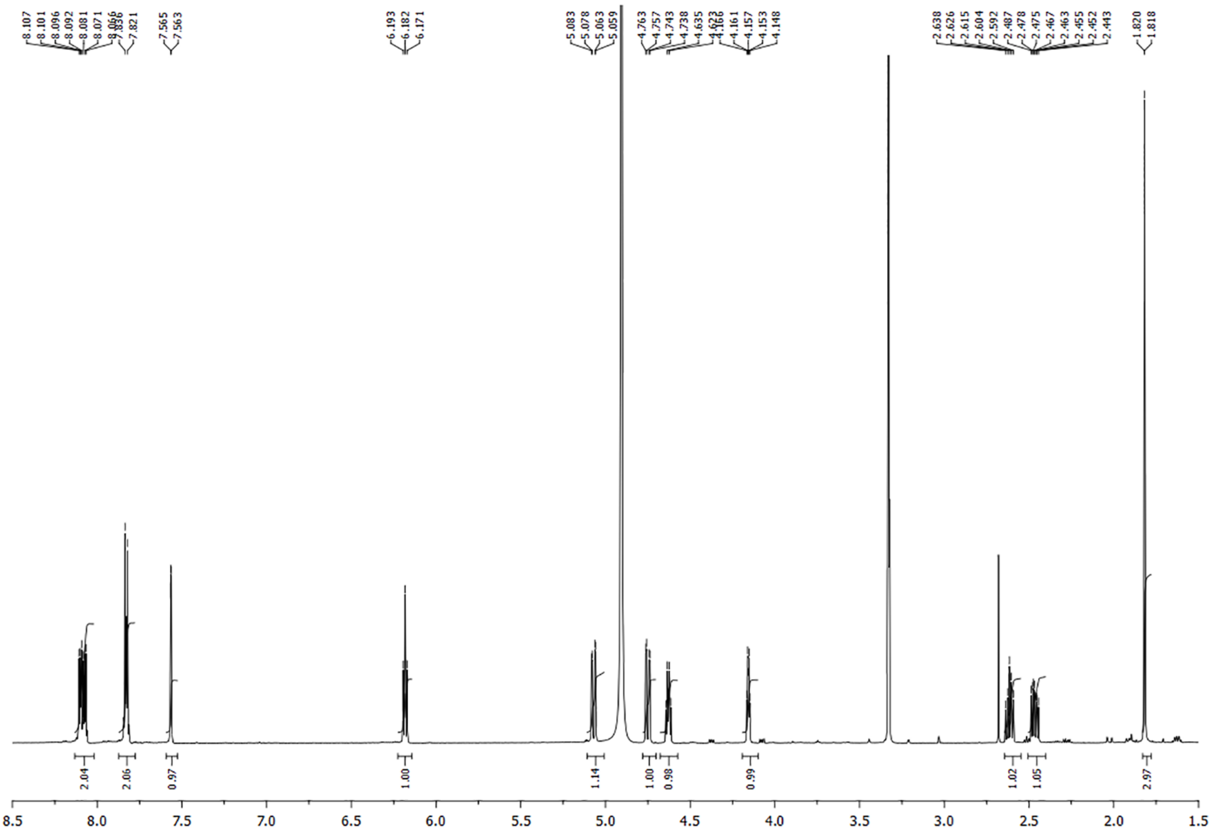

Figure S3. The ^{1}H NMR spectrum (600 MHz, CD_3OD) of 2-chloro-3-(3’-azide-3’-deoxythymidine)-1,4-naphthoquinone **5**.

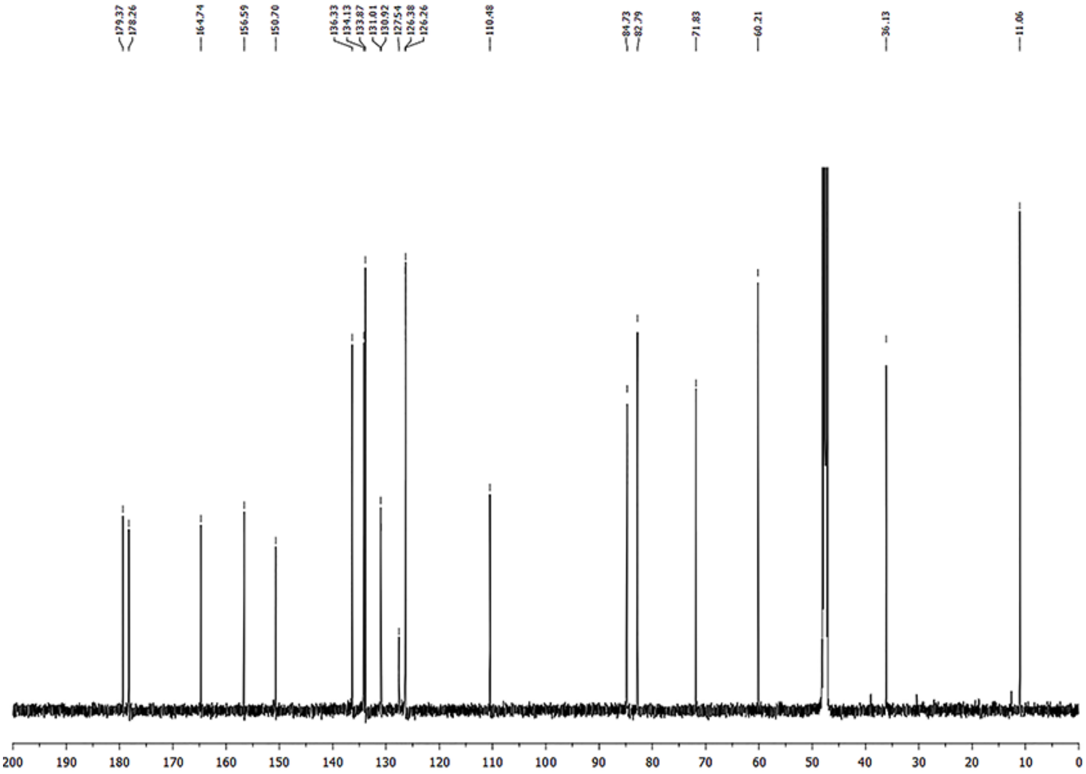

Figure S4. The 13C NMR spectrum (150 MHz, CD3OD) of 2-chloro-3-(3’-azide-3’-deoxythymidine)-1,4-naphthoquinone 5.

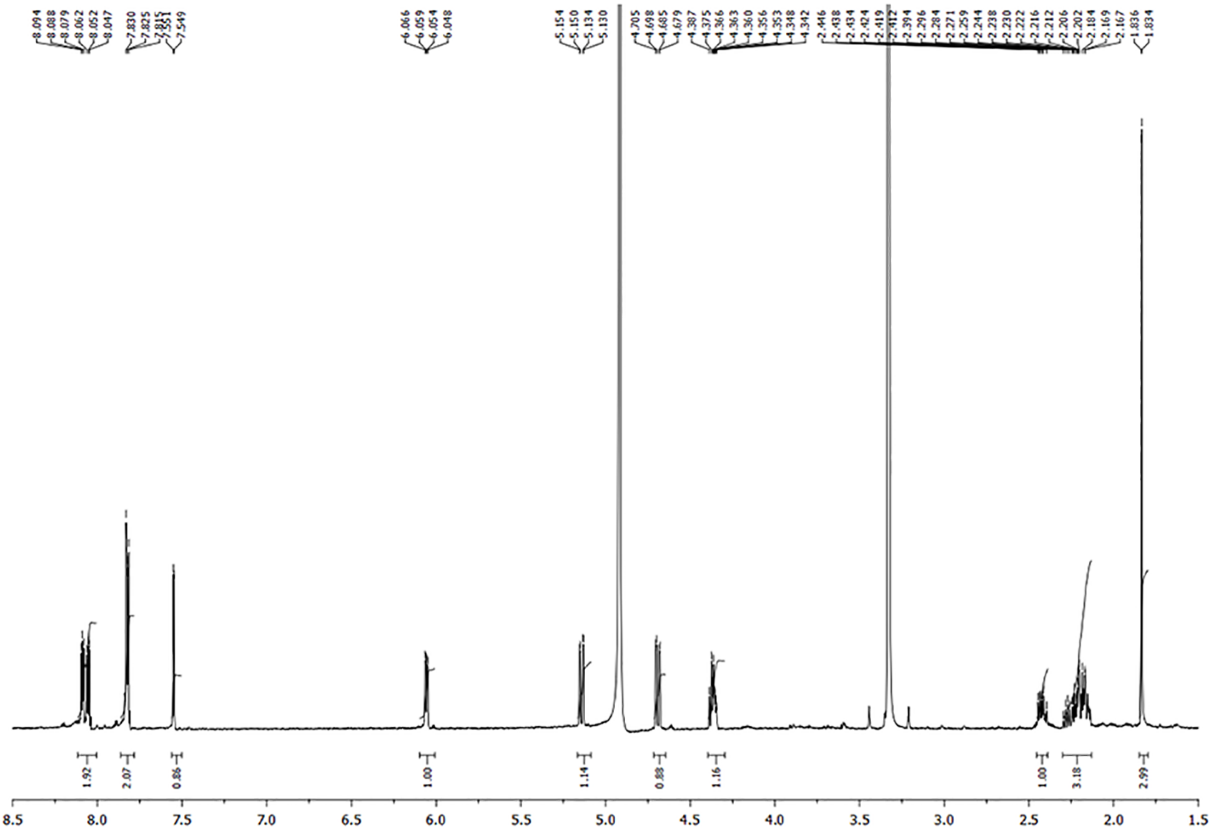

Figure S5. The 1H NMR spectrum (600 MHz, CD3OD) of 2-chloro-3-(3’-deoxythymidine)-1,4-naphthoquinone 6.

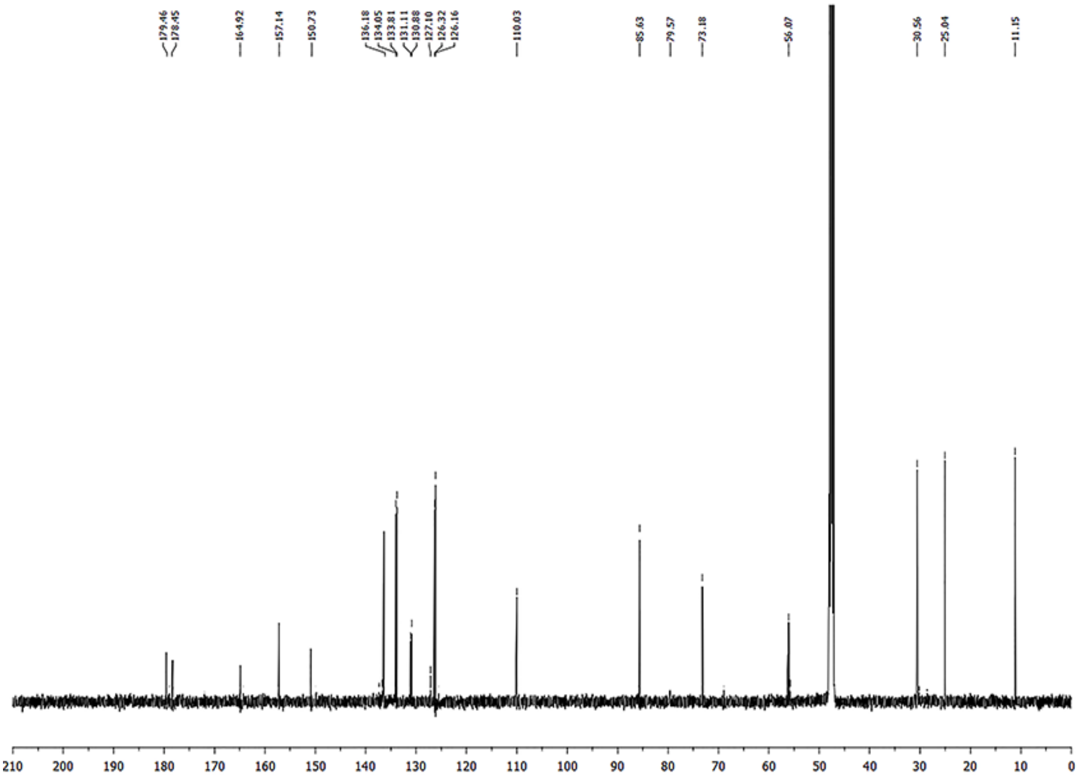

Figure S6. The 13C NMR spectrum (150 MHz, CD3OD) of 2-chloro-3-(3’-deoxythymidine)-1,4-naphthoquinone 6.

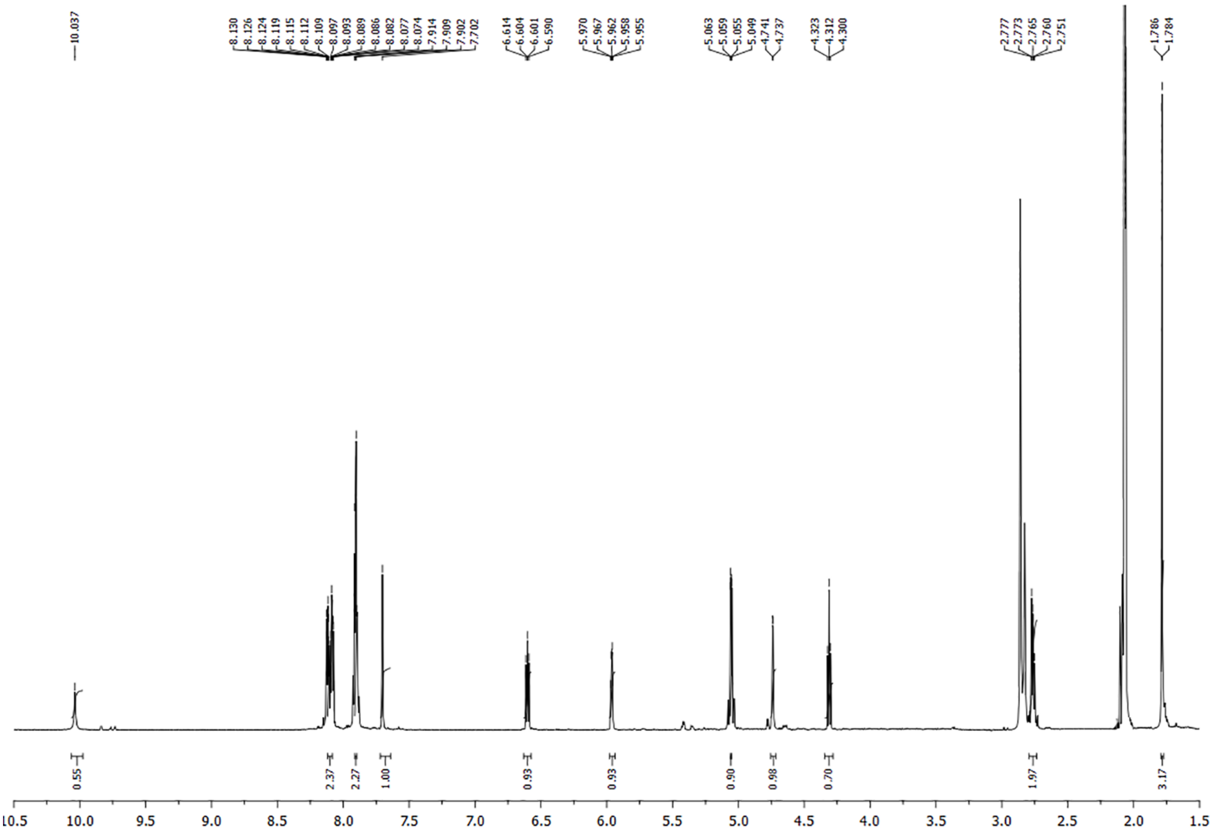

Figure S7: The 1H NMR spectrum (600 MHz, aceton-d6) of 2-chloro-3-thymidine-1,4-naphthoquinone 7.

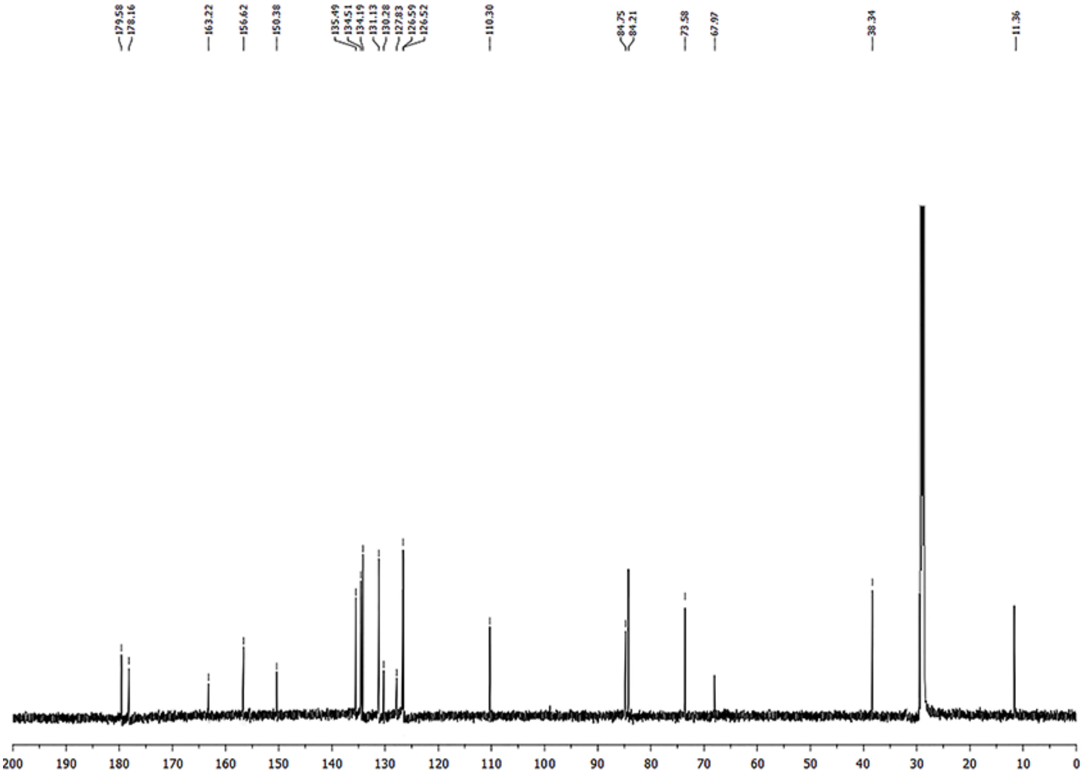

Figure S8.The 13C NMR spectrum (150 MHz, aceton-d6) of 2-chloro-3-thymidine-1,4-naphthoquinone 7.
